# Effects of Brain-Derived Neurotrophic Factor on Local Inflammation in Experimental Stroke of Rat

**DOI:** 10.1155/2010/372423

**Published:** 2011-02-24

**Authors:** Yongjun Jiang, Ning Wei, Juehua Zhu, Tingting Lu, Zhaoyao Chen, Gelin Xu, Xinfeng Liu

**Affiliations:** Department of Neurology, Jinling Hospital, School of Medicine, Nanjing University, 305 East Zhongshan Road, Nanjing 210002, China

## Abstract

This study was aimed to investigate whether brain-derived neurotrophic factor (BDNF) can modulate local cerebral inflammation in ischemic stroke. Rats were subjected to ischemia by occluding the right middle cerebral artery (MCAO) for 2 hours. Rats were randomized as control, BDNF, and antibody groups. The local inflammation was evaluated on cellular, cytokine, and transcription factor levels with immunofluorescence, enzyme-linked immunosorbent assay, real-time qPCR, and electrophoretic mobility shift assay, respectively. Exogenous BDNF significantly improved motor-sensory, sensorimotor function, and vestibulomotor function, while BDNF did not decrease the infarct volume. Exogenous BDNF increased the number of both activated and phagocytotic microglia in brain. BDNF upregulated interleukin10 and its mRNA expression, while downregulated tumor necrosis factor *α* and its mRNA expression. BDNF also increased DNA-binding activity of nuclear factor-kappa B. BDNF antibody, which blocked the activity of endogenous BDNF, showed the opposite effect of exogenous BDNF. Our data indicated that BDNF may modulate local inflammation in ischemic brain tissues on the cellular, cytokine, and transcription factor levels.

## 1. Introduction

Stroke is a major cause of death and long-term disability worldwide [1, 2]. Brain-derived neurotrophic factor (BDNF) can decrease infarct volume and improve neurological outcome either by exogenously supplied or overexpression in *vivo* using genetic methods in experimental stroke. Inhibition of BDNF exaggerates damage of ischemia. BDNF exerts neuron protection against ischemic injury through binding to two membrane receptors, p75 neurotrophin receptor and tyrosine kinase receptor B (trkB) [3]. 7,8-dihydroxyflavone as a bioactive high-affinity TrkB agonist also protects neurons from apoptosis and decreases infarct volumes in animal model of stroke [4]. 

Inflammation plays an essential role in the pathogenesis of ischemic stroke [5, 6]. Rapid activation of resident inflammatory cells (mostly microglia), productions of inflammatory cytokines such as interlerukin10 (IL-10) and tumor necrosis factor *α* (TNF-*α*) and translocation of intercellular transcription factors such as nuclear factor-kappa B (NF-*κ*B) are characters of local inflammatory responses to ischemia in brain [7–11]. Different responses may have different functions in the pathogenesis of stroke. Activated microglia could excrete neurotrophic effects such as BDNF to alleviate ischemic injury and exhibit phagocytic activity disposing of degenerating elements [7, 11]. There are several cytokines involved in inflammatory process. TNF-*α*, as an important proinflammatory cytokine, appears to exacerbate cerebral injury of ischemia [8] while IL-10, an anti-inflammatory cytokine, ameliorates ischemic insult of brain [9]. Activation of NF-*κ*B, an important transcription factor, could mediate translation of many downstream genes and promote survival of neurons [10]. 

BDNF promotes cell proliferation, increases phagocytic activity and inhibits apoptosis of microglia in brain [12]. BDNF downregulates the expression of TNF-*α* and upregulates the expression of IL10 in the model of multiple sclerosis [13]. NF-*κ*B activated by BDNF protects cells from damages, such as the serum starvation and glutamate toxicity [14]. However, whether BDNF modulates inflammatory processes in ischemic stroke is unclear. In present study, we evaluated the effect of BDNF on local cerebral inflammatory process on cellular, cytokine and transcription factor levels in ischemic stroke.

## 2. Methods

### 2.1. Animals

All protocols were approved by the Animal Care Committee (Institute of Science and Technology, Jiangsu Province, China). All procedures were performed under the guideline published in the NIH guide for the Care and Use of Laboratory Animals (National Institutes of Health Publication no. 85-23, revised 1985). Adult male Sprague-Dawley rats (220–250 g) were provided by Model Animal Research Centre of Jinling Hospital (Nanjing, Jiangsu, China). The rats were housed under controlled environmental conditions with ambient temperature of 25°C, relative humidity of 65%, and 12/12-h light-dark cycle. Food and water were provided *ad libitum*. All efforts were made to minimize the number of animals used and their sufferings.

### 2.2. Experimental Groups

The rats used in the study were randomized into control (*n* = 18), only vehicle was given, BDNF group (*n* = 18), BDNF was given, and antibody group (*n* = 18), BDNF antibody was induced. The procedure was shown in Table 1.

### 2.3. Administration of Drugs

Rats were anesthetized with pentobarbital sodium (40 mg/kg i.p., Sigma-Aldrich, USA) and placed in a stereotaxic frame. Stereotaxic injections were made by Hamilton syringe using the following coordinates: 0.5 mm rostral to bregma, 3.5 mm lateral to midline, and 5.5 mm ventral to the skull surface. Rats in control group were given 10 *μ*L phosphate buffered saline (PBS, pH 7.4); rats in antibody group were given 5 *μ*g BDNF antibody (PeproTech, USA) diluted in 10 *μ*L PBS (pH 7.4); rats in BDNF group were given 10 *μ*g BDNF (PeproTech, USA) diluted in 10 *μ*L PBS (pH 7.4). At the end of injection, the needle was left in place for 5 minutes before being slowly withdrawn.

### 2.4. Transient Middle Cerebral Artery Occlusion (MCAO) and Reperfusion

The MCAO procedure has been described before [15]. In brief, rats were anesthetized with pentobarbital sodium (40 mg/kg i.p.). Carotid artery was exposed and a 4-0 silicone-coated nylon filament was gently advanced from external carotid artery into the lumen of internal carotid artery until the rounded tip blocked the origin of the middle cerebral artery. A laser Doppler flow meter (LDF; Perimed PF5000, Stockholm, Sweden) was used to confirm the decrease of the middle cerebral artery blood flow immediately after the occlusion to about 20% of the basic cerebral blood flow. After 2 hours, rats were briefly reanesthetized and the filament withdrawn. Rectal temperature was maintained at 37°C using a rectal probe and heating pad during the surgery. Physiological parameters were monitored pre- and during ischemia (Table 2).

### 2.5. Tissue Processing

Rats were sacrificed at 6 h and 24 h of reperfusion. Rats were deeply anesthetized. For real-time quantitative PCR, enzyme-linked immunosorbent assay and electrophoretic mobility shift assay, rats were perfused transcardially with 0.1 M PBS (pH 7.4) only, and brains were removed rapidly and stored in the liquid nitrogen until used; for immunofluorescence, rats were perfused transcardially with 0.1 M PBS (pH 7.4) followed by a fixative solution containing 4% paraformaldehyde in PBS (pH 7.4). Brains were removed and fixed in the same fixative solution for an additional 6–12 h in 4°C. Prior to cytosectioning, tissues were cryoprotected using 20% sucrose in PB for 24 h followed 30% sucrose in PB for 48 h.

### 2.6. 2,3,5-Triphenyltetrazolium Chloride (TTC) and Terminal Deoxynucleotidyl Transferase-Mediated dUTP End-Labeling (TUNEL) Assay

For TTC staining, five coronal sections were made from the bremega to the cerebellum and stained with 2% TTC (Sigma-Aldrich, USA) at 37°C for 30 minutes. The infarct area of each section was measured in a blinded manner using Image J (NIH, USA). The infarct volume was then calculated by Swanson's method [16]. To determine apoptosis-like cell death, TUNEL staining (In Situ Cell Death Detection Kit, POD; Roche, USA) was performed 24 h after reperfusion (*n* = 6). The brain slices were mounted onto slides, fixed 20 minutes with 4% paraformaldehyde in PBS (pH 7.4), and pretreated with 3% H_2_O_2_ in methanol and 0.1% Triton X-100. The TdT enzyme and nucleotide mix were then added at proportions specified by the kit for 60 minutes at 37°C The slides were washed 3 times with PBS (pH 7.4). 50 *μ*L horse-radish peroxidase (POD) was added and slides were incubated in a humidified chamber for 30 minutes at 37°C. After washed 3 times with PBS (pH 7.4), 75 *μ*L diaminobenzidine (DAB) was added. Slides were incubated for 10 minutes at room temperature. The slides were mounted under glass coverslip and analyzed under light microscope.

### 2.7. Behavioral Testing

Following recovery from anesthesia, behavioral neurologic deficits were assessed 24 h after MCAO. The battery consisted of four tests to systematically evaluate motor, sensory, and vestibulomotor deficits. The following behavioral tests were performed. Postural reflex and hemiparesis test as described before [17]: (0) no observable neurologic deficits, (1) left forepaw flexion, (2) decreased resistance to lateral push and forepaw flexion without circling, (3) decreased resistance to lateral push and forepaw flexion with circling, and (4) cannot walk spontaneously. 

Forepaw placing test was done as described previously [18]. For each test, limb placing scores were as follows: (0) immediate and complete placing, (1) delayed and/or incomplete (>2 s), and (2) no placing. 

Modified beam balance test was done as described [19]: The scale was as follows: (1) steady posture with paws on top of the beam, (2) paws on side of the beam or wavering, (3) one or two lim(s) Slip off, (4) three limbs slip off, (5) attempts with paws on the beam, but falls, and (6) drapes over the beam, then falls or falls with no attempt. 

Adhesive tape test was done as before [20]: The scale was evaluated as: (1) <10 seconds; (2) 10–19 seconds; (3) 20–29 seconds; (4) 30–39 seconds; (5) 40–49 seconds; (6) 50–59 seconds; (7) ≥60 seconds.

### 2.8. Immunofluorescence

All sections (7 *μ*m) were simultaneously run to ensure identical staining conditions. After rinsing in PBS (pH 7.4), sections were incubated at 37°C for 2 h with the primary antibodies in PBS (OX-42 in pH 7.4, ED1 in pH 7.2) used at the following dilutions: OX-42 (1/200), ED-1 (1/200) (AbD Serotec, UK). After 4 washes, antibody visualization was achieved by the incubation at 37°C for 30 minutes with Alexa Fluor 488-conjugated donkey antimouse (1/200) (Invitrogen, USA). Negative controls were prepared by omitting the primary antibodies. Sections were then coverslipped with a fluorescent mounting medium (Sigma-Aldrich, USA). Sections were stored at 4°C until viewing. Sections were viewed under a Leica SP5 confocal microscope (Leica, France).

### 2.9. Enzyme-Linked Immunosorbent Assay (ELISA)

At 6 h and 24 h of reperfusion, 6 rats of each group were sacrificed and brain homogenates were obtained from the ischemic hemisphere. The concentrations of BDNF, IL-10 and TNF-*α* in brain homogenates were measured using specific ELISA kits according to the manufacturers' instructions (R&D system, USA).

### 2.10. Real-Time Quantitative PCR

Total RNA was isolated from frozen brain tissues using the TRIzol reagent (Invitrogen, USA) according to the manufacturer's recommendation, and subjected to DNase (Promega, USA) treatment. Reverse transcription (RT) reaction was carried out using the Firststrand cDNA synthesis kit (Takara, Japan) according to the manufacturer's instructions. Obtained cDNA were amplified using the following primers: for TNF-*α*, 5′-GCATGATCCGAGATGTGGAA-3′ and 5′-AGACACCGCCTGGAGTTCTG-3′, for IL-10, 5′-CCTTACTGCAGGACTTTAAGGGTTA-3′ and 5′-CTGGGCCATGGTTCTCT-3′, and for *β*-actin, 5′-GACAGGATGCAGAAGGAGATTACT-3′ and 5′-TGATCCACATCTGCTGGAAGGT-3′. The amplification and data acquisition were run on a real time PCR system (Bio-Rad, USA) using SYBR green PCR Master Mix (Takara, Japan). The conditions were predenaturation at 95°C for 10 minutes, followed by 40 cycles at 95°C for 15 seconds and 55°C for 1 minute. All samples were analysed in triplicates in three independent experiments. Reactions without cDNA were used as no template control and no RT controls were also set up to rule out genomic DNA contamination. Gene expression levels were calculated using the 2^−ddCt^ method [21].

### 2.11. Electrophoretic Mobility Shift Assay (EMSA)

Nuclear extracts were prepared by hypotonic lysis followed by high salt extraction. EMSA was performed using a kit (Gel Shift Assay System, Promega, USA) to assay NF-*κ*B DNA-binding activities. The NF-*κ*B oligonucleotide probe, 5′-AGTTGAGGGGAC TTTCCCAGGC-3′, was end-labeled with [*γ*-^32^P]. Protein-DNA binding assays were performed with 50 *μ*g of nuclear protein. The binding medium contained 4% glycerol, 1% NP40, 1 mM MgCl_2_, 50 mM NaCl, 0.5 mM EDTA, 0.5 mM DTT, and 10 mM Tris/HCl (pH 7.5). In each reaction, 20000cpm of a radiolabeled probe was included. Samples were incubated at room temperature for 15 minutes, and nuclear protein with ^32^P-labeled oligonucleotide complex was separated from free ^32^P-labeled oligonucleotide by electrophoresis through a 4% native polyacrylamide gel in 0.5 × TBE. After separation was achieved, the gel was dried (80°C, 30 minutes) and exposed to X-ray film (Fuji Hyperfilm) at −80°C with an intensifying screed.

### 2.12. Cell Counting

Images from brain sections with immunofluorescence were captured and analyzed by using Image-Pro Plus 6.0. Cells were counted in the ischemic cortex. 5 slides, 2 mm interval from bregma, were used for counting in each rat. 6 random areas of each slide were involved and the average number of OX-42 or ED1-positive cells/mm^2^ was calculated.

### 2.13. Statistical Analysis

The data were presented as mean ± SD. Differences between groups were compared using analysis of variance (ANOVA) followed by post hoc *t*-test with SPSS13.0 software (USA). *P* < .05 was considered to be statistically significant.

## 3. Results

### 3.1. BDNF Level was Increased in Brain

**Figure 1:**
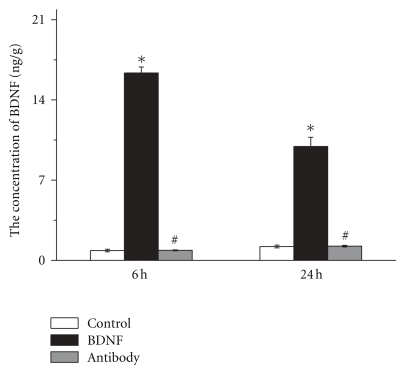
The concentration of BDNF in brain one hour before ischemia, 5 *μ*g of BDNF antibody in 10 *μ*L PBS was injected to the brain of rats. 10 *μ*g BDNF in 10 *μ*L PBS was given immediately after ischemia. At 6 h and 24 h of reperfusion, rats were sacrificed and brains were removed. The concentration of BDNF was measured after reperfusion using ELISA kits. The level of BDNF was significantly increased in BDNF group both 6 h and 24 h after reperfusion. Bars represent mean ± SD (*n* = 6). *P < .05 versus control group, ^#^ *P* < .05 versus BDNF group.

To assess the concentration of BDNF in brain tissues, the brain homogenates were obtained 6 h and 24 h after reperfusion and the concentration of BDNF was measured using ELISA kits. BDNF level was significantly increased by 19.7 fold in BDNF group while administration of BDNF antibody did not influence BDNF level in brain (1.00 ± 0.05 versus 0.98 ± 0.13 ng/g, *n* = 6, *P* > .05, Figure 1). The concentration of BDNF was decreased at 24 h of reperfusion than that at 6 h of reperfusion; however, it was significantly increased compared with control group (11.36 ± 0.91 versus 1.42 ± 0.08 ng/g, *n* = 6, *P* < .05, Figure 1). There was no significant difference of BDNF level between antibody group and control group (1.38 ± 0.14 versus 1.42 ± 0.08 ng/g, *n* = 6, *P* > .05, Figure 1).

#### 3.1.1. BDNF Protected Brain against Ischemic Insult and Reduced Neurologic Deficits

**Figure 2:**
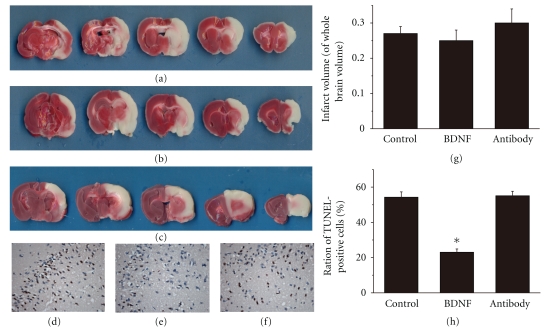
BDNF alleviated cellular injury of ischemic insult brain tissue sections obtained from injured cerebral hemispheres were stained with triphenyltetrazolium chloride (TTC) and TUNEL assay 24 h after reperfusion. (a) Infarct volume without any treatment. (b) Infarct volume with exogenous BDNF. (c) Infarct volume with exogenous BDNF antibody. (d) TUNEL assay in control group. (e) TUNEL assay in BDNF group. (f) TUNEL assay in antibody group. (g) No significant difference of infarct volume in the three groups. (h) Summary of TUNEL-positive cells 24 h after ischemia. Scar bar: 100 *μ*m. Bars represent mean ± SD (*n* = 6); *P < .05 versus control group.

We investigated cellular injury using TUNEL assay and measured the infarct volume using the TTC staining while neurologic deficits were tested by four behavioral tests 24 h after reperfusion. The number of TUNEL-positive cells in BDNF group was significantly decreased than that in control group (*n* = 6, 23.05 ± 1.86 versus 54.26 ± 3.05%, *P* < .05, Figures 2(d), 2(e), 2(h)) while BDNF antibody did not increased the TUNEL positive cells (*n* = 6, 55.06 ± 2.57 versus 54.26 ± 3.05%, *P* > .05, Figures 2(d), 2(f), 2(h)). However, the infarct volume measurements were found to be similar in the three groups with no statistical differences. Exogenous BDNF significantly improved motor-sensory function (1.5 ± 0.55 versus 2.8 ± 0.41, *P* < .05, Table 3), sensorimotor function (3.8 ± 0.98 versus 5.2 ± 0.75, *P* < .05, Table 3), vestibulomotor function (2.2 ± 0.98 versus 4.2 ± 0.75, *P* < .05, Table 3) compared with control group while BDNF antibody did not change the functions above. There was no significant difference of somatosensory function in the three groups (Table 3).

### 3.2. BDNF Increased the Number of Activated Microglia in Brain

Activated microglia could be marked by OX-42 and detected by immunofluorescence. BDNF increased the number of activated microglia compared to control group (394.7 ± 31.5 versus 272.0 ± 22.6/mm^2^, *n* = 6, *P* < .05, Figure 2) as early as 6 h after reperfusion. On the other side, when endogenous BDNF's activity was suppressed by BDNF antibody, the number of activated microglia was significantly decreased (90.7 ± 19.4 versus 272.0 ± 22.6/mm^2^, *n* = 6, *P* < .05, Figure 2). At 24 h of reperfusion, more microglia were activated in BDNF group than in control group (408.0 ± 22.1 versus 277.3 ± 21.9/mm^2^, *n* = 6, *P* < .05, Figure 2). However, there was no significant difference of the number of activated microglia between antibody group and control group (266.7 ± 24.1 versus 277.3 ± 21.9/mm^2^, *n* = 6, *P* > .05, Figure 2).

### 3.3. BDNF Increased the Number of Phagocytotic Microglia in Brain

**Figure 3:**
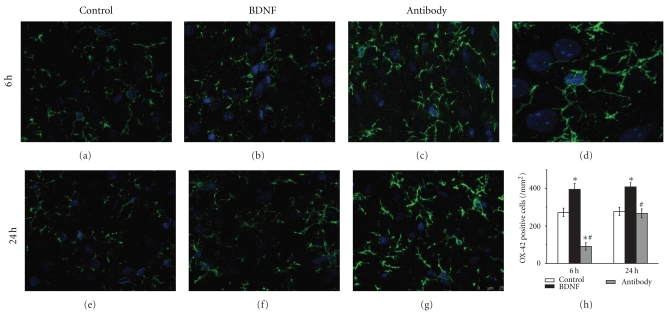
BDNF increasing the number of activated microglia after stroke Activated microglia in brain was marked by OX-42 and detected by confocal microscope following immunofluorescence. (a–c) 6 h after reperfusion and (e–g) 24 h after reperfusion; (a, e) control group, (b, f) BDNF group and (c, g) antibody group. (h) The number of phagocytotic microglia in BDNF group was significantly increased than control group 6 h and 24 h after reperfusion. BDNF antibody significantly decreased the number of activated microglia 6 h after reperfusion. Bars represent mean ± SD (*n* = 6). *P < .05 versus control group, ^#^ *P* < .05 versus BDNF group. Scale bars: 25 *μ*m.

ED1 is a marker of phagocytotic microglia which could engulf damaged cells and other inflammatory cells. The number of phagocytotic microglia in brain was significantly increased in BDNF group than in control group 6 h after reperfusion (378.7 ± 13.1 versus 237.3 ± 18.7/mm^2^, *n* = 6, *P* < .05, Figure 3). BDNF antibody, which could blocked the effect of BDNF, decreased the number of ED1 positive microglia (82.7 ± 12.0 versus 237.3 ± 18.7/mm^2^, *n* = 6, *P* < .05, Figure 3) 6 h after reperfusion. At 24 h of reperfusion, exogenous BDNF increased the number of phagocytotic microglia (325.3 ± 19.4 versus 138.7 ± 16.5/mm^2^, *n* = 6, *P* < .05, Figure 3). However, the number of ED1 positive microglia showed no significant difference in antibody group compared with control group (154.2 ± 26.5 versus 138.7 ± 16.5/mm^2^, *n* = 6, *P* > .05, Figure 3).

### 3.4. BDNF Promoted Anti-Inflammatory Cytokine Expression

**Figure 4:**
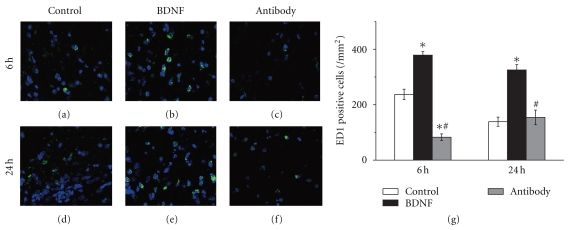
BDNF upregulating the number of phagocytotic microglia after stroke ED1 is well known as a marker of phagocytotic microglia. (a–c) 6 h after reperfusion and (d–f) 24 h after reperfusion; (a, d) control group, (b, e) BDNF group and (c, f) antibody group. BDNF significantly increased the number of phagocytotic microglia in brain 6 h and 24 h after reperfusion. The number of activated microglia was decreased in BDNF antibody group at 6 h of reperfusion. Bars represent mean ± SD (*n* = 6). *P < .05 versus control group, ^#^ *P* < .05 versus BDNF group. Scale bars: 25 *μ*m.

**Figure 5:**
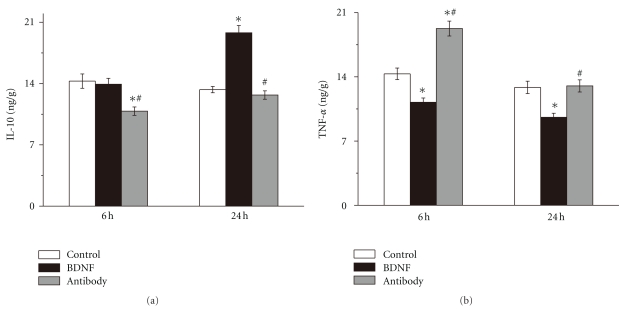
BDNF modulating local cytokine in brain after stroke Interleukin10 (IL10) and tumor necrosis factor *α* (TNF-*α*) in rat brain after stroke were measured using ELISA kits. (a) Exogenous BDNF upregulated IL-10 24 h after reperfusion while BDNF antibody decreased IL-10 6 h after reperfusion. (b) Exogenous BDNF decreased TNF-*α* at 6 h and 24 h of reperfusion than control group and BDNF antibody overwhelmed the effect. Bars represent mean ± SD (*n* = 6). *P < .05 versus control group, ^#^ *P* < .05 versus BDNF group.

IL10 is a well-known anti-inflammatory cytokine. IL-10 and its mRNA expression were tested by ELISA kits and real time qPCR, respectively. When BDNF antibody blocked activity of endogenous BDNF in brain, IL10 was markedly decreased compared to control group (10.85 ± 0.48 versus 14.28 ± 0.82 ng/g, *n* = 6, *P* < .05, Figure 4(a)). Application of BDNF antibody also prevented upregulation of IL10 mRNA 6 h after reperfusion (0.50 ± 0.02 versus 1.01 ± 0.09, *n* = 6, *P* < .05, Figure 5(a)). However, there was no significant difference of IL10 and its mRNA expression between BDNF group and control group 6 h after reperfusion. Exogenous BDNF markedly increased local IL10 level in brain tissues 24 h after reperfusion (19.80 ± 0.83 versus 13.31 ± 0.36 ng/g, *n* = 6, *P* < .05, Figure 4(a)) and increased mRNA expression by 1.93 fold compared with control group. However, BDNF antibody did not influence the level of IL10 and its mRNA 24 h after reperfusion.

### 3.5. BDNF Inhibited Proinflammatory Cytokine Expression

TNF-*α* is a proinflammatory cytokine. To determine whether BDNF inhibited local TNF-*α* in ischemic brain tissues, we used ELISA kits and real-time qRCR to measure TNF-*α* and its mRNA expression, respectively. Giving exogenous BDNF inhibited TNF-*α* (11.23 ± 0.46 versus 14.33 ± 0.63 ng/g, *n* = 6, *P* < .05, Figure 4(b)) while suppression of endogenous BDNF by BDNF antibody upregulated TNF-*α* (19.76 ± 0.81 versus 14.33 ± 0.63 ng/g, *n* = 6, *P* < .05, Figure 4(b)) 6 h after reperfusion. Exogenous BDNF also significantly decreased TNF-*α* (9.59 ± 0.44 versus 12.85 ± 0.67 ng/g, *n* = 6, *P* < .05, Figure 4(b)) at 24 h of reperfusion. On mRNA level, BDNF inhibited mRNA expression of brain TNF-*α* after stroke at both 6 h (0.39 ± 0.01 versus 1.00 ± 0.06, *n* = 6, *P* < .05, Figure 5(b)) and 24 h (0.77 ± 0.04 versus 0.91 ± 0.06, *n* = 6, *P* < .05, Figure 5(b)) of reperfusion. The mRNA of TNF-*α* was increased by 2.28 fold in antibody group 6 h after reperfusion. However, BDNF antibody did not influence the TNF-*α* and its mRNA 24 h after reperfusion.

### 3.6. BDNF Increased DNA-Binding Activity of NF-*κ*B after Stroke

**Figure 6:**
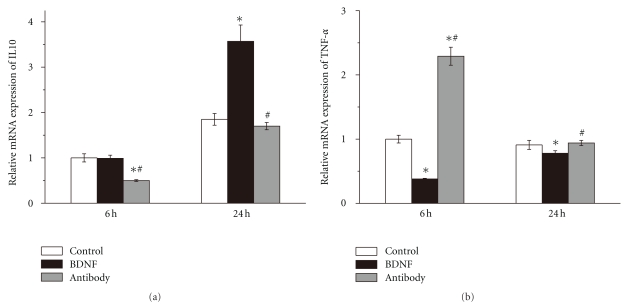
Effect of BDNF on mRNA expression of cytokine brain homogenates were obtained from ischemic cortexes 6 h and 24 h after reperfusion. The expressions of mRNA of interleukin10 (IL10) and tumor necrosis factor *α* (TNF-*α*) in rat brain after stroke were measured by real-time quantitative polymerase chain reaction (PCR). (a) Levels of IL10 mRNA. Application of BDNF antibody prevented upregulation of IL10 mRNA 6 h after reperfusion. At 24 h of reperfusion, BDNF increased level of mRNA of IL10 by 1.93 fold. (b) Levels if TNF-*α* mRNA. Exogenous BDNF significantly decreased mRNA of TNF-*α* 6 h and 24 h after reperfusion. Inhibition of endogenous BDNF significantly increased the expression of TNF-*α* 6 h after reperfusion. Bars represent mean ± SD (*n* = 6). *P < .05 versus control group, ^#^ *P* < .05 versus BDNF group.

The DNA-binding activity of NF-*κ*B was activated by many stresses such as ischemia, the activity was measured using EMSA and expressed as arbitrary densitometric units (AU). Exogenous BDNF increased DNA binding activity of NF-*κ*B by 10.7% compared with control group 6 h after reperfusion, and when endogenous BDNF was suppressed by BDNF antibody, the activity was significantly decreased (35.92 ± 0.99 versus 39.97 ± 0.70, *n* = 6, *P* < .05, Figure 6). At 24 h of reperfusion, exogenous BDNF could significantly increase DNA bind activity compared with control group (45.38 ± 0.86 versus 42.46 ± 0.27, *n* = 6, *P* < .05, Figure 6). However, there is no significant difference between antibody group and control group (41.43 ± 1.18 versus 42.46 ± 0.27, *n* = 6, *P* > .05, Figure 6).

## 4. Discussion

Our data suggested that BDNF could alleviate cellular injury of ischemic insult, reduce the neurologic deficits and modulate local inflammation on cellular, cytokine and nuclear factor levels in brain after stroke. Exogenous BDNF could increase the number of activated and phagocytotic microglia, upregulate IL-10, downregulate TNF-*α* and increase the DNA-binding activity of NF-*κ*B while BDNF antibody blocked these effects of BDNF in ischemic brain tissues. 

Firstly, we showed that introducing BDNF directly to brain increased the concentration of BDNF. We also found that BDNF antibody may not change the expression of BDNF in brain tissues after stroke. Our data confirm previous reports that injection of BDNF directly to brain could raise concentration of BDNF.

Secondly, exogenous BDNF could protect brain from ischemic injury and reduce the neurologic deficits. We found exogenous BDNF significantly decreased the number of TUNEL-positive cells. We also found that BDNF improved the motor-sensory function, sensorimotor function, and vestibulomotor function. Our results were consistent with previous reports [22, 23]. However, BDNF may not reduce the infarct volume which was different from some previous researches [3, 24]. Schäbitz et al. [22] also found that BDNF may not change the infarct volume.

Thirdly, we provided evidences that BDNF modulated local inflammation in ischemic brain tissues on cellular, cytokine and transcript levels. On cellular level of local inflammation, we found that exogenous BDNF could increase the number of both activated and phagocytotic microglia as early as 6 h after reperfusion and lasted at least for 24 h. When the activity of endogenous BDNF was blocked by BDNF antibody, the number of activated and phagocytotic microglia was decreased. Previous reports showed that BDNF could promote microglial proliferation and phagocytic activity in *vitro *and in *vivo* and inhibit microglial apoptosis [11, 12]. These results were consistent with our data. The mechanism BDNF activates microglia might be that BDNF could sustain elevation of intracellular Ca^2+^ of microglia [25, 26]. It is well accepted that activated microglia protects the brain against ischemic and excitotoxic injury [27]. Once microglia activated, it could excrete neurotrophins such as BDNF. BDNF could protect neurons against ischemia. More BDNF could activate more microglia and it is an autoloop. Phagocytotic microglia could engulf damaged cells and other inflammatory cells which may initiate more damage to brain in ischemic stroke. These may partly explain the protection of BDNF against ischemic insult. 

On cytokine level of brain local inflammation in ischemic stroke, we found that BDNF could decrease brain TNF-*α* in ischemic stroke. Exogenous administration of BDNF decreased brain TNF-*α* and inhibited the mRNA expression of TNF-*α*. When the activity of BDNF was blocked by its antibody, the protein level and mRNA expression of local TNF-*α* in brain were decreased. These results were consistent with previous research, which shows that exogenous BDNF inhibits the expression of TNF-*α* in mouse brain of model of multiple sclerosis [13]. TNF-*α*, an important proinflammatory cytokine, plays a vital role in the pathophysiology of ischemia stroke [6]. Exogenous administration of TNF-*α* exacerbates ischemic brain injury while inhibition of TNF-*α* could reduce brain damage [8, 28]. BDNF could provide protection of brain in ischemic stroke via decreasing local TNF-*α*. 

BDNF not only decreased local proinflammatory cytokine, it also increased local anti-inflammatory cytokine. IL10 is an important anti-inflammatory cytokine. Our data showed BDNF increased the level of IL10 and upregulated the mRNA expression of IL10 at 24 h of reperfusion. Once activity of endogenous BDNF was blocked by BDNF antibody, local IL-10 and its mRNA in brain were increased. These results were consistent with other studies [13]. Previous publications showed that exogenous pre/postischemic administration of IL10 can provide neuroprotection following MCAO [9, 29]. Over-expression of IL10 in *vivo* markedly protected cortical tissue against cerebral ischemia using the IL10 transgene mice [30]. Our data suggested that BDNF might protect brain from ischemia through upregulating local IL10 in brain.

On the transcription level of local inflammation in brain in ischemic stroke of rats, we found that exogenous BDNF increased the DNA-binding activity of NF-*κ*B. We also provided evidence that the DNA-binding activity of NF-*κ*B was inhibited when the effect of BDNF was suppressed. These results indicated that BDNF could modulate NF-*κ*B's activity. The way BDNF activates NF-*κ*B in different cells is through the TrkB-PI3-kinase-Akt pathway [31]. Inhibition of TrkB, the receptor of BDNF in the cell, decreases the activation of NF-*κ*B [32]. The activation of NF-*κ*B induced by BDNF protects cells from a variety of damages, including the serum starvation, glutamate toxicity and ischemia [14]. Once the activity of NF-*κ*B was inhibited by different inhibitors, the protections of BDNF against different damages were lessened [31]. Our data suggested that NF-*κ*B played an important role in neuron protection of BDNF.

In our study, effect of BDNF on local inflammation in brain showed no significant difference between antibody group and control group 24 h after reperfusion. This may because that BDNF antibody only blocked the activity of BDNF and may not suppressed the expression of local BDNF in brain after stroke. Our data showed that BDNF antibody did not change BDNF level (Figure 1). 24 h after reperfusion (25 h after BDNF antibody was given), the expression of new BDNF may replace the antibody-conjuncted BDNF, so the effect of BDNF antibody might be removed.

## 5. Conclusions

In summary, our data suggested that BDNF may alleviate cellular injury of ischemic insult, reduce the neurologic deficits and modulate local inflammation on cellular level, cytokine level, and transcription factor level in ischemic stroke.

##  Conflict of Interests

The authors have no conflict of interests to disclosure.

##  Authors' Contributions

Y. Jiang, N. Wei, and X. Liu participated in concept and design of the study, acquisition of raw data, analysis and interpretation of data, drafting manuscript, critical revision of the manuscript for scientific validity, statistical analysis. J. Zhu, T. Lu, Z. Chen and G. Xu participated in study concept and design, acquisition of raw data and critical revisions of the paper. Y. Jiang and N. Wei contributed equally to this work.

## Figures and Tables

**Figure 7:**
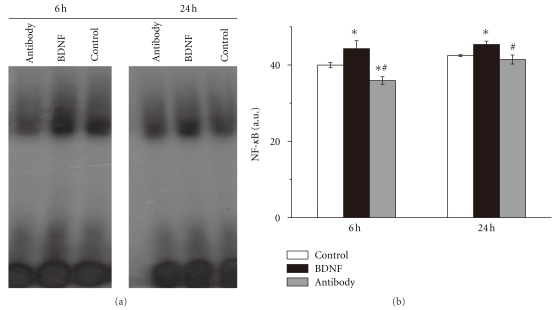
BDNF enhancing DNA-binding activity of NF-*κ*B The DNA-binding activity of NF-*κ*B in ischemic cortex in three groups was measured using Electrophoretic mobility shift assay (EMSA) 6 h and 24 h after reperfusion. Levels of NF*κ*B DNA binding activity were expressed as arbitrary densitometric units (AU.) using Image J. Exogenous BDNF increased the activity of NF-*κ*B 6 h and 24 h after reperfusion. Inhibition of endogenous BDNF by its antibody significantly decreased the activity 6 h after reperfusion. Bars represent mean ± SD (*n* = 6). *P < .05 versus control group, ^#^ *P* < .05 versus BDNF group.

**Table 1:** Procedure of the experiment.

Group	*n*	−3 h	−2 h	0 h	6 h	24 h
Control	18	PBS	MCAO & PBS	Reperfusion	Sacrifice	Sacrifice
BDNF	18	PBS	MCAO & BDNF	Reperfusion	Sacrifice	Sacrifice
Antibody	18	Ab	MCAO & PBS	Reperfusion	Sacrifice	Sacrifice

PBS, phosphate buffered saline; MCAO, middle carotid artery occlusion; BDNF, brain-derived neurotrophic factor; Ab, BDNF antibody. “−”, before reperfusion.

**Table 2:** Physiological parameters per- and during ischemia.

Group		pH	PaCO_2_ (mmHg)	PaO_2_ (mmHg)	MABP (mmHg)	T(°C)
Control	pre	7.45 ± 0.02	36.5 ± 1.9	132.0 ± 13.0	74.8 ± 13.1	37.1 ± 0.33
	during	7.43 ± 0.02	39.5 ± 3.4	123.3 ± 20.1	81.2 ± 10.9	36.9 ± 0.30
BDNF	pre	7.45 ± 0.01	37.8 ± 3.3	119.2 ± 13.1	77.3 ± 11.9	37.0 ± 0.34
	during	7.42 ± 0.01	40.5 ± 4.4	140.3 ± 20.1	85.2 ± 11.6	37.2 ± 0.50
Antibody	pre	7.45 ± 0.02	36.4 ± 3.7	139.8 ± 16.7	74.0 ± 14.7	36.9 ± 0.34
	during	7.45 ± 0.01	40.2 ± 3.9	124.8 ± 11.5	78.6 ± 15.2	37.1 ± 0.40

Pre, preischemia; during, during ischemia (1 hour after onset of MCAO); pH, arterial pH; PaCO_2_, arterial carbon dioxide partial pressure; PaO_2_, arterial oxygen partial pressure; MABP, mean arterial blood pressure; T, rectal temperature.

**Table 3:** BDNF-reducing neurologic deficits.

	Postural reflex and hemiparesis test	Forepaw placing test	Modified beam balance test	Adhesive tape test	
				Remove	Contact
Control	2.8 ± 0.41	5.2 ± 0.75	4.2 ± 0.75	5.7 ± 0.82	4.5 ± 1.05
BDNF	1.5 ± 0.55*	3.8 ± 0.98*	2.2 ± 0.98*	4.3 ± 1.21	4.8 ± 0.75
Antibody	3.2 ± 0.75	5.5 ± 1.05	4.2 ± 0.98	5.8 ± 1.33	4.5 ± 0.55

*P < .05 versus control group.
